# Supplementing Closed Ecological Life Support Systems with In-Situ Resources on the Moon

**DOI:** 10.3390/life11080770

**Published:** 2021-07-30

**Authors:** Alex Ellery

**Affiliations:** Department of Mechanical & Aerospace Engineering, Carleton University, 1125 Colonel By Drive, Ottawa, ON K1S 5B6, Canada; aellery@mae.carleton.ca; Tel.: +1-613-520-2600 (ext. 1027)

**Keywords:** bioregenerative life support, closed ecological life support, in-situ resource utilization, lunar industrial ecology

## Abstract

In this review, I explore a broad-based view of technologies for supporting human activities on the Moon and, where appropriate, Mars. Primarily, I assess the state of life support systems technology beginning with physicochemical processes, waste processing, bioregenerative methods, food production systems and the robotics and advanced biological technologies that support the latter. We observe that the Moon possesses in-situ resources but that these resources are of limited value in closed ecological life support systems (CELSS)—indeed, CELSS technology is most mature in recycling water and oxygen, the two resources that are abundant on the Moon. This places a premium on developing CELSS that recycle other elements that are rarified on the Moon including C and N in particular but also other elements such as P, S and K which might be challenging to extract from local resources. Although we focus on closed loop ecological life support systems, we also consider related technologies that involve the application of biological organisms to bioregenerative medical technologies and bioregenerative approaches to industrial activity on the Moon as potential future developments.

## 1. Introduction

On Earth, human life is supported by a complex and deep biosphere with material recycling including hydrological and biogeochemical processes through the lithosphere, hydrosphere, cryosphere, atmosphere and biosphere. The key features of natural ecosystems are bio-material turnover and energy flows [1]. They are closed to matter permitted by material recycling through biogeochemical (CNPSK) cycles but open to energy from the Sun. Buckminster Fuller characterised the Earth’s biosphere as spaceship Earth in his *Operating Manual for Spaceship Earth* (1968). Artificial life support systems generally lack the large buffering capacity of the Earth’s biosphere so they require much higher degrees of precision control.

Biosphere 2 was a 12,700 m^2^ glass biospheric enclosure sealed with silicone sealant in the Arizona desert housing a crew of 8 people for two years (1991–1993) with effectively 100% material closure [2]. Energy was input to Biosphere 2 as solar energy and electric generators supplying 700 kW (average) to 1500 kW (peak). The biosphere included 7 modules of 1900 m^2^ tropical rainforest, 1300 m^2^ savanna, 1400 m^2^ desert, 450 m^2^ tidal (freshwater and saltwater) marshes, 850 m^2^ ocean, 2500 m^2^ agricultural system and a 2400 m^2^ human habitat. The habitat comprised a galley, living quarters, an analytic laboratory, computing facilities, machine shop and sickbay facilities. A system of cooling water towers, chilled water and a water boiler-controlled Biosphere 2′s air temperature [3]. Biosphere 2 incorporated 6 × 10^6^ litres of water including fish/rice paddies and hosted 3800 different species including three domestic animals (pigmy goat, feral swine and chicken) which consumed inedible crop residue and worms in return for milk, eggs and tilapia meat. Waste was processed through composting and bacterial processing. Food production consumed the majority of the crew’s time. The facility incorporated two large expansion chambers (“lungs”) to accommodate temperature variations to ensure low gas leakage rates ~10%/year [4]. The most challenging issues were O_2_/CO_2_ level fluctuations which required periodic intervention and the calorie-restricted diet imposed on the crew. Obviously, the scale of Biosphere 2 renders it impractical for space application (except perhaps O’Neill colonies [5]). Nevertheless, experiments to date suggest that 100% closure is feasible for up to 6 months but the precise means to achieve this has yet to be demonstrated.

## 2. Role of Ecologies

Crucially, in space or planetary surfaces, we are transplanting the human from the environment in which we have evolved to an entirely alien one in which we have not. In this regard, it is crucial to consider evolutionary medicine as a factor in designing life support systems for long duration missions—diet (with implications for diabetes), microbiome (with implications for autoimmune disease), radiation exposure (with implications for cancer), infectious disease exposure (with implications for virulence), emotional isolation (with implications for mental disorders) [6].

Environmental control and life support systems (ECLSS) involve control of atmospheric pressure, temperature, humidity and composition with most other resources supplied. In the early days of spaceflight, life support systems stored oxygen, water and food for astronaut consumption and returned waste back to Earth. A more comprehensive life support system also requires: (i) air quality including the maintenance of buffering gases, CO_2_ removal and O_2_ generation; (ii) food production and storage; (iii) water management through waste-water recovery; and (iv) solid waste management through bacterial processing. There are several approaches to such life support: (i) open loop life support systems in which all consumables are supplied and stored as adopted in early space missions of short duration; (ii) physicochemical recycling life support systems that recycle bulk consumables; (iii) bioregenerative life support systems that exploit biological mechanisms; (iv) in-situ resource utilisation supply of consumed material; (v) hybrid life support systems that are combinations thereof.

**Figure 1:**
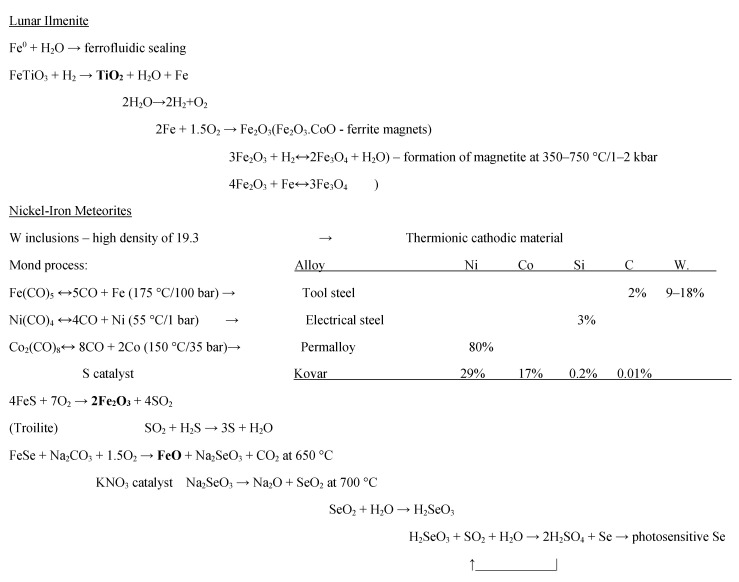

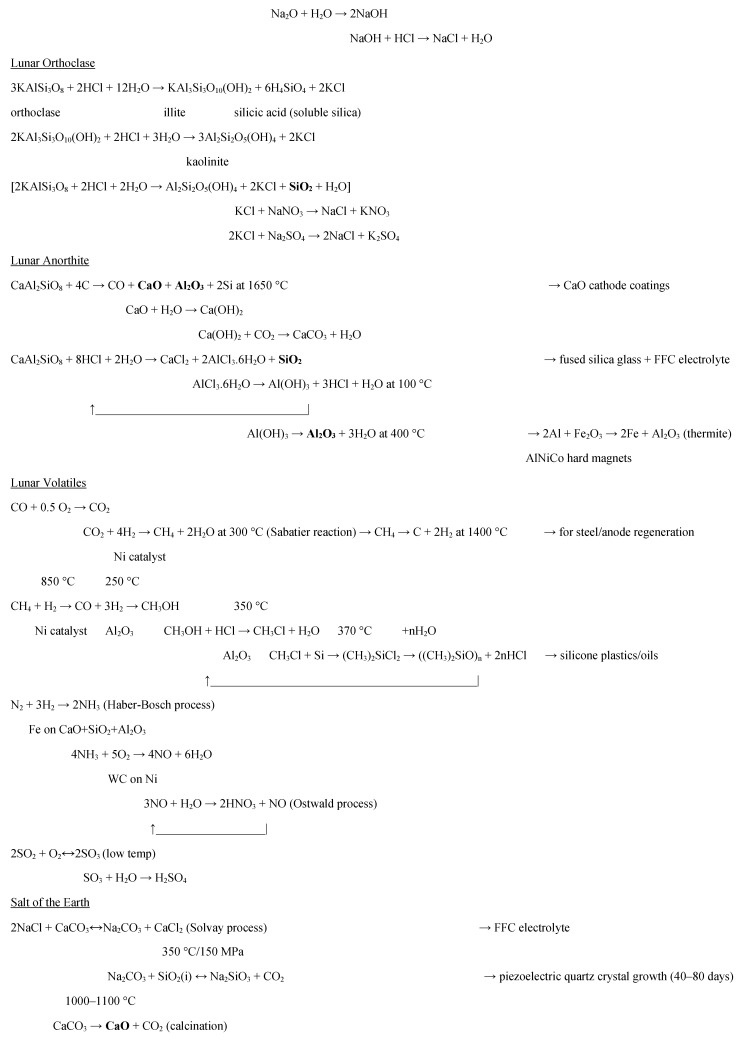

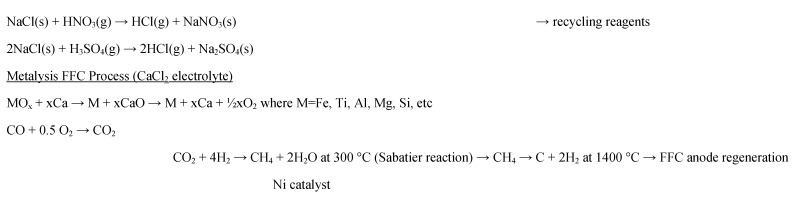
Near closed loop lunar industrial ecology (emboldened materials are pure metal oxides for direct reduction using the Metalysis FFC process). This summarises the sustainable closed loop lunar industrial ecology system (CLIES) presented in [9]. Energy generation and storage issues required to support CLIES are discussed in [12].

A human being requires 38.5 kg of consumables per day including 24.8 kg of water for showering, toilet flushing, cleaning and clothes washing per day including tankage. Water is the primary human consumption requirement—it is the main constituent of electrolytes of the human body (blood plasma, interstitial fluid and intracellular fluid) regulated by the kidneys through urine production [7]. Grey water is readily recycled so 13.7 kg of water consumables per day is more realistic. Recycling of water and oxygen may be implemented through physicochemical processes, the two components that can be readily sourced and supplied from lunar resources. Water at 5.6 ± 2.9% concentration (plus associated vapours of H_2_S, ethylene, CO_2_ and methanol) was detected by the LCROSS (lunar crater observation and sensing satellite) mission (2009) in an ejecta plume generated by a Centaur rocket stage impacting into the Cabeus crater [8] (Table 1). However, there are considerably greater resources in lunar regolith minerals which may be extracted through a handful of processes (Figure 1). The closed loop lunar industrial ecology system (CLIES) extracts other resources from lunar minerals and impacted asteroidal material [9]. Industrial ecology is an approach to organising industrial chemical processors such that the waste of one process becomes feedstock for another, i.e., recycling to minimize waste. CLIES presents an interlocking set of closed recycling loops that extract ceramics and ultimately metals from common rock-forming lunar minerals. Some of these metals might be exploited as input supplies to CELSS beyond water/oxygen. Scarce lunar volatiles may also be extracted but should serve only as recycled reagents so they are not consumed but their scarcity renders them untenable as a source of CNPS in CELSS. Similarly, CLIES does not exploit lunar volatiles for consumption. One notable exception was the carbon resources—this was proposed to manufacture silicone (siloxane) products as elastomeric electrical insulation plastic for wiring harnesses and silicone oils for lubrication [10] but we have re-addressed this issue and concluded that silicone is unnecessary as glass cloth and porcelain may be substituted for this purpose [11] and tungsten disulphide (WS_2_) is a high temperature lubricant used in place of molybdenum disulphide (MoS_2_) which may substitute for silicone oils. Hence, all extracted volatiles are recycled with CLIES. The implication is that, although H_2_O resources can be replenished from lunar resources, CNPS elements cannot be supplied from lunar volatile sources but must be recycled within CELSS. However, CLIES can supply a limited set of resources for input to CELSS but the majority of nutrients including most metal micro-nutrients specified later must be recycled within CELSS. Water and oxygen supplies are available on the Moon but current proposals for mining local water ice are focused on its use as cryogenic propellant/oxidiser. We proffer a view that extraction and consumption of water consumables for burning as propellant/oxidiser wastes finite and valuable resources which would otherwise support human survival on the Moon over future generations.

Material closure and openness to energy flow fundamental facets of any closed loop biospheric ecology with the latter driving it to far-from-equilibrium conditions [13]. So, it is with life support systems—material closure (with the exception of water/oxygen) will be essential. However, closed loop food production and nutrient recovery from waste can only be provided by biological processes employing living organisms in plant cultivation which is volume intensive. Closed biological regeneration involves the production of food, recycling of waste, recycling of water and air regeneration.

## 3. Physicochemical Processes

The modular approach to life support avoids centralisation of life support with ventilation ducting across the modules. Each module operates on a stand-alone basis without intermodule ducting as each has its own independent power and life support systems. This approach also provides inherent safety to astronauts. However, such ducting aids in air circulation. In a lunar base, standard atmospheric pressure of 101 kPa may be reduced as long as the oxygen partial pressure is maintained at 20 kPa but no greater than 48 kPa (beyond which oxygen toxicity is induced) but control of flammability imposes a lower ceiling of no more than 30 kPa. Reducing the proportion of buffer gases increases fire risk (though EVA suits adopt 100% oxygen to reduce internal pressure which retards suit flexibility). Nitrogen is required for atmospheric buffering and agriculture but it is a scarce commodity on the Moon. The major biological elements required to support human life—CHONPSK—must, excepting H and O, be supplied from Earth or recycled efficiently as they are scarce resources on the Moon. Required macronutrients also include metals—3.5 g/day K, 2.5 g/day Na, 1 g/day Ca, 260 mg/day Mg, 14 mg/day Fe, 7 mg/day Zn and 1.5 mg/day P supplemented by micronutrients Mn, Cu, Zn, Sn, Mo, Pb, Al, Ti, B, Ni, Cr, V and Co though excess Ni, Co and Cr are toxic. Nevertheless, micronutrients are essential [14], e.g., Keshan disease is a juvenile cardiomyopathy common in Se-deficient areas of China; Mo deficiency is apparent only in conjunction with excess W intake; sufficient Cr is necessary for insulin sensitivity. While water is an abundant resource on the Moon, K and P requires complex extraction from KREEP (potassium-rare earth-phosphorus) minerals and C, N and S volatile resources are highly rarefied. Trace elements may be more problematic though Fe, Ca, Co and Se can be sourced from asteroidal material on the Moon. Other trace elements may be resident in lunar regolith in small quantities but extraction will be challenging. It is essential to minimise the resupply of consumables from Earth implying that extensive recycling will be necessary.

A human being in a single day consumes 641 g dry food, 3216 g water (approximately 50% drinking water and 50% water associated with food) and 806 g oxygen while excreting 94 g faeces, 1630 g urine and 943 g carbon dioxide [15]. Water is also required for washing of the body for hygiene (7270 g), dishes (5460 g), clothing (12,500 g) and flushing (500 g) [16]. Hence, water is by far the dominant resources consumed. Recycling of oxygen and water permit elimination of 90% of the consumable supply to support human life. To date, most approaches to recycling are physicochemical involving the recycling of air and water with food being re-supplied and waste stored and/or dumped. Yet water and oxygen are potentially supplied from in-situ resources on the Moon. On the Moon, oxygen may be extracted from ilmenite using H_2_ (FeTiO_3_ + H_2_ → Fe + TiO_2_ + H_2_O with recycling of H_2_ through H_2_O → H_2_ + ½O_2_), CO (FeTiO_3_ + CO → Fe + TiO_2_ + CO_2_ with recycling of CO through 2CO_2_ → 2CO + O_2_) or CH_4_ (FeTiO_3_ + CH_4_ → Fe + TiO_2_ + CO + 2H_2_ with recycling of CH_4_ with 2CO + 6H_2_ → 2CH_4_ + 2H_2_O) reducing agents, hydrogen being the obvious choice given the apparent availability of water ice on the Moon. There may be several reservoirs of lunar water [17]: (i) as subsurface regolith ice; (ii) as thin films on minerals; (iii) as water of hydration in minerals; (iv) within mineral inclusions.

ECLSS refers to all life support systems functioning to control the environment to support human life—there is no specific implication of closing loops but partial recycling with varying degrees of some consumables has been achieved. The International Space Station (ISS) ECLSS system provided several specific functions integrated in two ECLSS subsystems, the oxygen generation system (OGS) and the water recovery system (WRS) which recycle water and oxygen at 70–80% [18,19] so requires some resupply:

1. pumping cabin air between modules with motorised fans;
2. maintaining cabin air temperature at 22 °C using heat exchangers;
3. monitoring and controlling total atmospheric pressure and the partial pressures of N_2_, O_2_ and CO_2_ in cabin air;
4. monitoring and controlling the water vapour content of cabin air (humidity at 40%) using desiccants such as silica (which can be sourced on the Moon);
5. mass spectrometer for analysing aerosols, particles, water vapour and gases in the air to provide analytical feedback;
6. fire, smoke and CO detection and suppression;
7. high efficiency particulate air (HEPA) filtering of solid particles from cabin air using replaceable filters impregnated with biocides to prevent microbial infection;
8. airborne contaminant removal such as methane (CH_4_) and ethylene (C_2_H_4_) from cabin air using activated charcoal beds;
9. CO_2_ extracted from cabin air using LiOH (2LiOH + CO_2_ → Li_2_CO_3_ + H_2_O) granules in canisters integrated with the charcoal beds. Alternatively, a molecular sieve such as zeolite (sodium, potassium or calcium aluminosilicate) can remove CO_2_ in air. Zeolite is manufactured through hydrothermal synthesis—they are formed by slow crystallisation of heated aqueous solutions of SiO_2_ and Al_2_O_3_ (both of which can be sourced from lunar resources) in NaOH. A membrane commonly used for recovering CO_2_ from the atmosphere is PDMS (polydimethylsiloxane) silicone rubber because of its high permeability to CO_2_ relative to other gases (PDMS is manufacturable from syngas through the Rochow process). In these cases, CO_2_ is removed without recycling. This may be employed as backup to recycling mechanisms or recycling mechanisms added. The Bosch reaction at 550–700 °C catalytically reduces CO_2_: CO_2_ + 2H_2_ → C + H_2_O. The catalyst is activated steel wool—it is only 10% efficient, far lower in efficiency than the Sabatier reaction. The Sabatier reactor catalytically reduces CO_2_ with H_2_ from the OGS to generate CH_4_ and H_2_O at 180–550 °C: CO_2_ + 4H_2_ → CH_4_ + 2H_2_O. This is exothermic with 98% efficiency. The catalyst is typically ruthenium-on-alumina and the methane is vented but on the Moon, it should be stored as a carbon source. Water electrolysis is required to recycle H_2_ and release O_2_ as the oxidant to CH_4_ fuel or for recycling CO_2_ into O_2_ as part of ECLSS;
10. OGS generates O_2_ into cabin air by electrolysing water from the WRS with H_2_ vented or passed to the Sabatier reactor which gives 98% recovery. In both Bosch and Sabatier reactors, water is then electrolysed into its constituents to recycle H_2_ for the Bosch or Sabatier reaction and yield O_2_: 2H_2_O → 2H_2_ + O_2_. There are several water electrolysis methods. Suitable solid-state electrolytes include calcia-stabilised zirconia or yttria-doped ceria at 850 °C with an electrical energy consumption of 250 W. Static feed water electrolysis electrolyses water using an aqueous KOH electrolyte soaked onto thin asbestos sheets. Solid polymer water electrolysis uses a solid polymer membrane electrolyte of perfluorinated sulphonic acid polymer.
11. WRS reclaims wastewater, urine and condensation through vacuum distillation followed by multifiltration beds giving 80% water recovery—it filters out solid particles initially and then filters out organic contaminants through semipermeable membranes and finally a catalytic oxidation reactor destroys volatile organic material and bacteria.

There are several extensions to such physicochemical processes that may be employed including the incorporation of fuel cells. The main types of fuel cell are polymer electrolyte membrane, alkaline, solid oxide, direct methanol and biological fuel cells. They all operate with hydrogen gas except the methanol fuel cell. Regenerative fuel cells require water electrolysis to split water into hydrogen and oxygen. Hydrogen and oxygen combustion releases an enthalpy of −285.8 kJ/mole H_2_/O_2_ at STP. Hydrogen and oxygen reactants for fuel cells is usually stored in the cryogenic liquid state. Rather than storing hydrogen cryogenically, it may be used for CO_2_ conversion and waste combustion [20]. CO_2_ conversion to oxygen uses hydrogen for the Sabatier reaction forming methane—methane may be cracked at high temperature >1000 °C to release H_2_ on demand and a graphite residue. Wastewater and urine may be recycled into pure water while simultaneously providing both thermal and electrical energy [21]. Aluminium powder (activated with 1–2.5% Li) reacts spontaneously with wastewater and/or urine at room temperature generating thermal energy at 23.5 MJ/kg of Al and hydrogen gas:

Al + 3H_2_O → Al(OH)_3_ + 3/2H_2_ + 420 kJ/mol

The hydrogen may be fed into a fuel cell generating electrical energy and freshwater. Polymer electrolyte membrane fuel cells may accommodate illuminated cultivation chambers supplied with oxygen and nutrients to support microalgae cultivation to recycle air through continuous photosynthesis and which may be harvested as food [22]. Exploitation of biological organisms in fuel cells constitutes microbial fuel cells. By way of illustrative example, microbial fuel cells have been employed as artificial metabolism onboard a small mobile robot (EcoBot) to permit it to engage in pulsed phototactic behaviour [23]. Microbial fuel cells exploit *Escherichia coli* in a bioelectrochemical medium to convert biochemical energy into electrical energy through a proton exchange membrane. The *E. coli* is fed with sugar at the anode which transfers electrons to it carried by the coenzyme NADH. The cathode balances the redox reaction. The pulsing behaviour was imposed by the low energy extracted from the microbial fuel cell. Biological fuel cells have very low power densities ~1 mW/cm^2^ compared with the methanol fuel cell at ~60 mW/cm^2^ and polymer electrolyte membrane fuel cell at 300–400 mW/cm^2^ rendering them an inefficient approach to energy storage.

## 4. Waste Processing

Lack of waste recycling will quickly lead to the depletion of certain elements such as N, K, Na, S, P, etc. On the ISS, urine, wastewater and water condensation is filtered and recycled into potable water. This represents a highly restrictive form of waste processing for the recovery of water only. Waste treatment is commonly conducted in quartz reactors, a quartz tube offering high temperature tolerance by virtue of its very low coefficient of thermal expansion. Organic waste constitutes human waste (urine and faeces) and inedible plant matter (such as cellulose, lignin, etc), the latter being relevant for in-situ food production. Typically, inedible plant matter is produced at 10 times greater dry weight than human faeces production. High-fibre plant waste (which may be as high as 90% of the crop) must be recycled either physicochemically through oxidation to carbon dioxide or biologically to improve processing efficiency. For wheat, there is a 20–40% loss from inedible plant material but inedible plant food can be fed to chickens and fish. Metabolic and plant waste cannot be used directly as manure for plant cultivation but must be composted first. This recycling of waste is a central tenet of permaculture. Recycling of solid and fluid human waste may be implemented through wet oxidative combustion in hydrogen peroxide to which an AC electric field is applied within a ceramic reactor [24]. This approach is rapid and suited to automatic control yielding waste gas and mineralised waste solution. Complete oxidation of hydrogen into water and hydrocarbons into waste gas requires a Pt catalyst—pressure measurement of waste gas production provides feedback on the state of the process. Mineralisation is regulated by alternating voltage control of the E-field electrodes. Wet oxidation must be coupled with nitrogen fixation. The mineralised waste product may be used as manure supplemented with Knop’s solution (for supplementary potassium) to grow crops such as wheat, peas and lettuce [25]. Full mineralisation of human waste is essential to prevent the proliferation of pathogenic bacteria [26]. Traditionally, the Haber-Bosch process has been used to fix nitrogen artificially by reacting nitrogen and hydrogen in the presence of a catalyst to form ammonia—the catalyst comprises a core of magnetite surrounded by a mantle of wustite and a shell of Fe with Al_2_O_3_ and CaO promoters all of which are derivable from lunar resources. Nitrogen fixation by rhizobium-infected legumes replenishes nitrate in the soil but this requires maintenance of a nitrogen buffer in the atmosphere. Recycling food and waste requires the adoption of bioregenerative methods. Hyperthermophilic aerobic bacteria may be employed for composting of human metabolic waste for use as agricultural fertiliser for forming lunar or Martian soil [27]. Bacterial fermentation generates temperatures up to 80–100 °C suitable for aerobic hyperthermophiles for decomposing waste yet sterilising pathogenic bacteria acting as a natural autoclave. The removal of NaCl from waste and back into the human recycling loop may be achieved through the cultivation of the edible salt-concentrating saltwort, *Salicornia europaea* or the alga *Spirulina* [28]. More conventionally, lettuce, celery, Chinese cabbage, Swiss chard, dill and radish accumulate high concentrations of NaCl from NaCl-supplemented Knop’s solution (equivalent to that of human urine) sufficient for 30 g of greens to support a low salt diet [29].

Water management systems are prone to biological fouling and mineral scaling in wastewater which can be physically filtered using granular lunar regolith [30]. Although evolutionary emergence and progression of viral disease is almost impossible to predict, epidemiological spread can be well modelled mathematically [31]. Microorganisms exhibit complex sociality in forming biofilms—implicated in up to 80% of human infections —as communal habitats of different microbial species embedded in a nutrient-rich extracellular matrix of DNA, protein and polysaccharides. Biofilms are crucial to the formation of fruiting bodies occurring under starvation conditions mediated by communication through quorum sensing [32]. Quorum sensing is used in bacteria to estimate their population density and regulate their behaviour collectively. They use quorum sensing to communicate and coordinate through extracellular chemical signals (pheromones) that activate the transcription of specific genes. Pheromones diffuse according to bacterial cell density so the production of public goods collectively is determined by the bacterial population. The marine bacterium *Vibrio fischeri* uses the pheromone AHL (N-acyl homoserine lactone) for quorum control of bioluminescence by affecting the transcription of two *lux* genes in neighbouring bacteria [33]. Similarly, both *Streptococcus pneumoniae* and *Staphylococcus aureus* use pheromones to activate genes for toxin production suggesting a means for controlling bacterial infections by inhibiting quorum sensing [34], e.g., degradation of AHL signals (quorum quenching) using lactonases and acylases. By detecting the concentration of specific acyl-homoserine lactone molecules, bacteria form biofilms, become virulent or develop antibiotic resistance. The biofilm provides protection to the microbes permitting communication, feeding and growth. The formation of biofilms—high density, structured colonies of bacteria embedded in an extracellular matrix—represent a bacterial strategy to restrict the invasion of inhibitor chemicals and exhibit enhanced resistance to antibiotics by preventing their infiltration through the extracellular matrix [35]. Similarly, bacterial swarming where bacteria migrate collectively exhibit swarm-specific resistance to antibiotics only while swarming. However, quorum sensing inhibitors degrade quorum sensing molecules to inhibit bacterial pathogenesis [36]. This can block virulence pathways to reduce toxicity of bacteria. Nitric oxide (NO) manufactured through the Ostwald process aids in dispersing bacterial biofilms reducing their pathogenic ability. This can reduce biological fouling and the risk of bacterial or toxic infection.

## 5. Bioregenerative Methods

The carbon loop, due to the scarcity of carbon on the Moon, cannot be recycled through physicochemical processes but the bioregenerative recycling loop has a long time constant. An agricultural system requires an infrastructure to support the growth of higher plants including providing a nutrient supply to roots, the recovery of water of transpiration (20 litre/day/m^2^) and provide a photosynthetically active radiation (PAR) lighting system [37,38]. Light may be supplied through solar collector mirrors to provide 0.5–1.0 kW/m^2^ of full spectrum sunlight. Fresnel lenses may also be used as solar concentrators to transmit light energy through optical fibres and distributed in a controlled manner that is independent of direct sunlight. However, during the lunar night, artificial lighting is essential. PAR may be supplied by kW-output lamps—high-pressure sodium lamps or fluorescent xenon lamps have been superseded by LEDs but they are limited in their light intensities for some plants such as spinach, tomato and bell pepper. However, sulphur-microwave lamps offer bright visible light with a near-solar spectrum—it comprises a quartz envelope filled with small amounts of S and Ar ionised by microwaves with high efficiency. Exposure to sunlight is also essential for the production of vitamin D for human health which may require vitamin supplementation during the lunar night. There is other life support hardware required including heat-generating motors, pumps, fans, etc with recirculating hydroponic fluid loops in the case of hydroponic agriculture. Environmental parameters must be monitored reliably and controlled for optimal growth. A system of distributed sensors is required to monitor temperature, fluid pressure, fluid pH, conductive or thermal moisture and electrochemical dissolved oxygen levels, e.g., MELiSSA (micro-ecological life support system alternative) compartments measure temperature, pO_2_ and solution pH. The implication is that such autonomous control must be robust to external perturbations, reliable without functional failure and stable to feedback time delays.

Closed ecological life support systems (CELSS) requires agricultural production for food, CO_2_ removal, O_2_ generation (human respiratory quotient of [CO_2_]/[O_2_] = 0.84–0.87 depending on the percentage formation of carbohydrate, fat and protein in the food consumed) and water recycling with bioreactors for recycling waste. Plants consume CO_2_ and H_2_O for photosynthesis under the action of sufficient PAR to produce carbohydrate food, regenerate oxygen and filter water through evapotranspiration. There have been several bioregenerative life support system programmes including Biosphere 2 (US), CELSS (NASA), Bios-3 (Roscosmos) and its predecessors and MELiSSA (ESA) [39]. CELSS require bioregenerative approaches which are characterised by significantly longer lags in recycling than physicochemical methods [40]. CELSS architectures are hierarchically modular, separating human habitation, plant cultivation, animal husbandry and microbial waste treatment which can be further subdivided [41]. They should be highly functionally redundant with multiple approaches to any specific function, i.e., there should always be physicochemical backup systems as far as possible [42]. A three-tiered architecture with planning–reactive-servo levels is considered suitable for controlling a complex life support system [43]. It is crucial to develop autonomous ecosystem control that includes mass and energy exchange measurements and models [39].

Crop growth rates may be modelled using the S-shaped Lotka-Volterra predator-prey logistics equations but their nonlinearities can give rise the chaotic behaviours [44]:

(1) $$\frac{dm_{in}}{dt}=r_{in}m_{in}\left(1-\frac{m_{in}}{m_{in}\left(f\right)}\right)$$

(2) $$\frac{dm_{ed}}{dt}=r_{ed}m_{in}\left(\frac{m_{ed}\left(0\right)+m_{ed}}{m_{ed}\left(f\right)}\right)\left(1-\frac{m_{ed}}{m_{ed}\left(f\right)}\right)$$

where *m_ed_* = edible biomass, *m_in_* = inedible biomass, *r* = growth rates, *m*(0) = initial (minimum) biomass, *m(f)* = final (maximum) biomass. This may be broken down into a mass flow model of growth of edible plant, growth of inedible plant, human consumption of edible plant, waste processing of inedible plant and waste processing of human waste [45].

ESA’s MELiSSA is a microorganism-based artificial ecosystem centred in Barcelona Spain to create a closed loop bioregenerative system for space application including microbial recycling of human waste. It exploits microbial bioreactors in which bacteria, yeast and algae can recycle all the major biochemical elements and degrade complex organic molecules in waste into usable materials. Microbial bioreactors with bacteria fixed to a filter bed can also act as biofilters to filter air. They are well-suited to carbon recycling in closed life support systems, e.g., cellulase degrades cellulose into its components such as edible glucose. MELiSSA comprises five (of which four are microbial) interconnected functional bioreactor compartments inspired by aquatic ecosystems with closed loop fluid flow [46,47,48]:

1. A multi-bacterial species anaerobic composter (including species from the complex human microbiome of which many bacterial strains resist culturing) that breaks down human and plant waste; it must also suppress methanogenesis (combusted methane imposes a loss of carbon and methane-consuming sulphate-reducing bacteria are sensitive to environmental conditions), e.g., *Fibrobacter succinogenes* is an anaerobic thermophilic bacteria whose fermentation degrades plant waste into CO_2_ and volatile fatty acids;
2. Stirred tank bioreactor with photoheterotrophic anaerobic bacteria (purple non-sulphur bacteria *Rhodobacteriaceae rubrum*) that absorbs fatty acid volatiles and converts them into edible biomass;
3. Packed bed reactor with immobilised aerobic nitrifying bacteria (involving two bacterial steps by *Nitrosomonas europaea* from NH_4_^+^ into NO_2_^−^ and *Nitrobacter winogradsky* from NO_2_^−^ into NO_3_^−^ that oxidises urea-produced ammonium NH_4_^+^ into nitrate NO_3_^−^ in a culture medium;
4. Gas-lift bioreactor with edible higher plant hydroponic system supplemented by edible cyanobacteria (*Arthrospira/Spirulina platensis*) in a culture medium for photosynthesis to generate food, purify water and recycle air;
5. Human habitation compartment.

MELiSSA is designed to produce 1 kg O_2_/person/day, 15.8 kg water/person/day, 2.7 kg food/person/day while removing 1.2 kg CO_2_/person/day [49]. Constraints on recycling of air, water and food include minimum mass, minimum logistics, minimum energy consumption of 170 W/person, minimum crew time, self-sufficiency and safety. The most challenging aspect has been microbial waste recycling—a six-person crew during a Mars mission of 216 days outbound, a surface sortie of 496 days and 216 days inbound generates waste comprising 5.50 tonnes CO_2_, 8.24 tonnes urine, 12.73 tonnes of non-recycled water [50]. The goal is to provide 100% recycling with mass balance for CHONPS cycles in a manner that is safe and robust [51]. The medium of bacterial culture comprises a mixture of variable amounts of components [52]: (i) freshwater algal cultural media comprises—NaNO_3_–MgSO_4_·7H_2_O–KH_2_PO_4_–NaOH–CaCl_2_·2H_2_O–NaCl–Al_2_(SO_4_)_3_·18H_2_O–Na_2_SiO_3_·9H_2_O–FeSO_4_·7H_2_O–EDTA–H_3_BO_3_–ZnSO_4_·7H_2_O–MnCl_2_·4H_2_O–Na_2_MoO_4_·5H_2_O–CuSO_4_·5H_2_O–Co(NO_3_)_2_·6H_2_O; (ii) saltwater algal cultural medium comprises–NaNO_3_–Na_2_HPO_4_·H_2_O–CuSO_4_·5H_2_O–ZnSO_4_·7H_2_O–CoCl_2_·6H_2_O–MnCl_2_·4H_2_O–Na_2_MoO_4_·5H_2_O. Although some of the metals of the culture medium could be sourced on the Moon, many and the volatiles cannot and require recycling biologically back into cultural media.

## 6. Food Production Systems

The introduction of food production is a key feature of the bioregenerative system—it also eliminates waste from discarded food packaging. One major consideration that has been exploited by crop breeders is that radiation environments can provide increased genetic mutation to breed hardier crops—the space environment increases mutation rates by 1% (compared with a terrestrial rate of 10^−4^%). However, such mutation rates are highly undesirable in a lunar production farm indicating that extensive shielding will be required and that piped sunlight will be necessary. Agriculture utilises natural photosynthesis of plants to convert human metabolic waste (CO_2_) combined with wastewater to yield O_2_ for human metabolism and renewable food sources: nCO_2_ + nH_2_O → (CH_2_O)_n_ + nO_2_. Only a small fraction of total water input to growing crops is required for photosynthesis; the vast majority can be recovered via evapotranspiration. Water is filtered through the roots passing up through the xylem within the stem out through the stomata underneath the leaves [53]. They can release 2–10 litres of water vapour/m^2^/day of leaf area by transpiration which can be exploited to purify wastewater. Higher plant crop area (m^2^) is determined by $A=\frac{M}{Y}$ where M = mass of edible crop/day, Y = nominal yield rate/m^2^/day. It has been suggested that biological recycling to support one human requires 20–40 m^2^ of agricultural land irradiated by 250–300 W/m^2^ of PAR and 10.8 kg/m^2^/day water to produce 1.25 kg of dried edible vegetation (to supply 3000 kcal/day) and 0.8 kg of inedible plant waste per day [54]. Water recycling driven by evaporation and condensation is crucial in closed ecological systems [55] though lunar water resources relax this requirement. Elevated temperatures, hydroponic nutrient delivery and high CO_2_ conditions may increase plant productivity, reduce water transpiration and reduce the required agricultural area to 10 m^2^/person [56,57]. An agricultural footprint of 10 m^2^ is sufficient to provide full O_2_ generation and 200% water requirement per person through photosynthesis but only 50% food requirement (assuming 20 m^2^ required for food production). This of course refers to food-producing land under cultivation. Human habitation requires additional square footage—the 315 m^2^ BIOS-3 facility comprised 4 compartments that sustained a human crew of 3 for 6 months with 100% recycling of air, 95% recycling of water and 50% recycling of food of which 25% was animal products [58].

Lunar regolith can be exploited for multiple roles including as an agricultural soil substrate for plant growth [59,60]. Lunar regolith comprises olivine, pyroxene and plagioclase feldspars with impact glasses and agglutinates. Clay byproducts from the artificial chemical weathering process in our lunar industrial ecology could provide a substrate for a clayey soil substrate (Figure 1). These clays may provide sources of Fe, Mg, Ca and K ions though they would be deficient in N, P, etc. On Mars, however, hydrated clays may have formed naturally early in the Noachian period (4.1–3.7 Gy ago) when the global basaltic crustal magma ocean reacted with the extremely dense outgassed steam [61]. The vast majority of Mars’ enormous early water equating to a global depth of 100–1500 m has been sequestered into water of hydration of crustal minerals through the Noachian period which without plate tectonic recycling remains sequestered [62]. Perchlorates in Martian soils are highly toxic but may be removed through heating: MgClO_4_ → MgO + Cl_2_ + 7/2O_2_.

Martian regolith appears to offer a more favourable soil substrate than lunar regolith even with impregnation with in-situ manufactured clays. However, inedible parts of plants may be recycled as compost for lunar regolith to create the humus component of soil. Metals such as Ni and Cr which occur in lunar regolith are toxic in excess to biology suggesting the use of soil-less hydroponics rather than lunar soils. Other extra-terrestrial regoliths may host agricultural soils. The Murchison and Allende carbonaceous chondrites are sources of C, N, S, P, Ca, Mg, Na, K and Fe [63]. They may be subjected to artificial weathering through hydrothermal processing to increase extraction rates of these elements. Mixed microbial cultures (though not higher plants) were successfully grown in Murchison meteorite samples in water [64]. Although carbonaceous chondrites have impacted continually over the aeons, the inventory of carbonaceous chondrite material on the Moon is very minor. The productivity of soil-based agriculture can approach that of hydroponics by enhancing light intensity with the advantage of simpler nutrient recycling [65]. It is crucial that methods such as no tilling and drip irrigation be adopted to prevent soil erosion and salinisation respectively, two of the central tenets of sustainable permaculture. Small animals may be bred such as worms which can grow on solid wastes within soil. High protein flour can also be obtained from dried worms: a 300-litre soil-bed can yield 60–80 kg of flour per year. Worms can provide food for fish which offer high food value. Experiments in cultivating plants such as tomato, wheat and cress in regolith simulants yielded superior growth in Martian regolith simulant over Moon regolith simulant but both were capable of supporting plant cultivation [66]. Moon regolith performance was most likely inferior due to aluminium but perchlorates were not incorporated into the Martian regolith simulant. Gravity-induced water flow rate of nutrients in soil was 90% lower under Martian gravity conditions (requiring 90% less water) than Earth but this did not increase solute resident time at the roots due to compensatory emission of nitrogen oxide gases (requiring less fertiliser for soil microorganism) [67]. Such effects may be accentuated under lunar gravity conditions.

Hydroponics and aeroponics offer advantages over soil cultivation with their high nutrient efficiency despite the higher water requirement in the case of the former—given the water resources on the Moon and Mars, this is not considered a major disadvantage. Hydroponics exploits a mineral nutrient solution directly to the exposed root system yielding 25% faster crop growth than soil culture. Seeds must germinate in a growing medium—the roots are supported by a porous inert material such as rockwool, perlite, vermiculite, arcillite and/or baked clay pellets. All are derivable from lunar resources. Perlite and vermiculate are superheated expanded volcanic glass materials with similar porosity to pumice. Arcillite is a calcined montmorillonite clay that is porous similar to vermiculite. Rockwool is the commonest growth medium comprised of basalt spun into bundles of fibres—a lunar version may be manufactured from lunar fibreglass. Although primarily for supporting seedlings, rockwool can be used throughout the plant lifecycle. The adoption of hydroponics is the default assumption of MELiSSA. Hydroponics permit indoor vertically-stacked rack-configured cultivation which may be integrated with structures [68]. Such vertical farming with hydroponics can yield food for a single person within a volume of 10–20 m^3^ compared with 400 m^3^ required in field agriculture but at the cost of higher energy consumption from 250 kWh/y/m^2^ to 3500 kWh/y/m^2^ primarily due to artificial lighting. There are several approaches to hydroponics—wick, deep-water culture, ebb-and-flow, drip method, nutrient-film technique and aeroponics [69]. Most suffer from clogging issues which is a significant problem. For minimum maintenance, the pipe-based ebb-and-flow technique involves few mechanical parts. Hydroponics requires aerated nutrient-rich water which must be recirculated. Knop’s hydroponic solution comprises the major inorganic components required to grow higher plants including 0.0144 mol calcium nitrate Ca(NO_3_)_2_, 0.0049 mol potassium nitrate (saltpeter) KNO_3_, 0.0145 mol magnesium sulphate MgSO_4_, 0.0130 mol potassium dihydrogen phosphate KH_2_PO_4_ and variable amounts of potassium chloride dissolved in water [70]. Most of these elemental components may be derived from lunar resources except for nitrogen, sulphur and phosphorous (and carbon) which must be recycled through CELSS, i.e., composting with saltpetre (potassium nitrate) as a fertiliser. Saltpeter may be converted to HNO_3_ with H_2_SO_4_ generating potassium bisulphate which decomposes to potassium sulphate at 100–120 °C:

KNO_3_ + H_2_SO_4_ → HNO_3_ + KHSO_4_

KCl + KHSO_4_ → HCl + K_2_SO_4_

K_2_SO_4_ may be stocked as a fertiliser for a stable source of potassium and sulphur. Generally, inorganic nutrient solutions can be supplemented with organic fertilisers such as processed animal manure, bonemeal, fishmeal, seaweed, dried insect flour, etc dissolved in water. Trees comprise a typical component of the terrestrial biosphere and in permaculture offer different layered niches for a diverse but compact ecological community—canopy (e.g., edibles leaves such as maple and mulberry), dappled layer (e.g., apples), shrub layer (e.g., berry bushes), herb layer (e.g., herbs), soil layer (e.g., wide variety of crops), rhizosphere (e.g., root vegetables), vertical climber layer (e.g., runner beans) and fungus layer (e.g., mushrooms). However, even dwarf varieties of trees are unsuited to CELSS due to their enormous bulk, deep rooting requirements and low yield of edible fruits, favouring bush-grown fruits such as berries.

A nominal complete terrestrial diet might include several basic foodstuffs: fish provide essential fatty acids; spinach provides a wide range of nutrients; carrots provide carotenoids (vitamin A); tomatoes provide lycopene; grapes provide resveratrol and antioxidants. However, fish and grapes present challenges. The salad machine is a conceptual device that produces 600 g of diverse edible produce per week (sufficient for a 50 g salad for a crew of 4 every other day) [71]. Most closed loop higher plant agricultural systems require around 20 crop species [72]. A wide range of plants are required to supply carbohydrates, protein and fats to support human metabolism [73] but dwarf varieties with high harvest index, high light and water efficiency, short growing cycle, high plant density, high nutrition and easy preparation are favoured [74]. While C4 photosynthesis comprises 2% of plant species, it accounts for 25% of global primary productivity on Earth. Staple crops, high in carbohydrates, include wheat and potato/sweet potato and other root vegetables such as onions, garlic, radish, carrots, beetroot and squash. Potato requires regular dark periods for the growth of tubers so would be unsuitable to 24 h lighting. Wheat is selected for its versatility and, like most crops, is a C3 plant but it can grow under continuous light [75]—although less efficient in photosynthesis than C4 plants like maize, they are more efficient at elevated CO_2_ levels. High protein sources include soyabean, pinto bean and peanut. Vegetables for micronutrients include tomato, bell pepper, chufa, chard, spinach, kale, cabbage, coriander and lettuce. Minerals are provided by bell pepper, lettuce, tomato, cabbage and strawberry. Sprouts such as soyabean and broccoli have high oxygen consumption until leaves sprout but silicate minerals in regolith offer an abundant oxygen source. A core daily diet of 100 g leafy greens (cabbage, spinach, lettuce, chard, etc), 100 g tomato, 70 g carrot and 50 g bell pepper is of particular importance in providing high nutrition [76,77]. Supply of sufficient vitamin D is particularly challenging for astronauts without supplements [78]. Glycophosphate is a common herbicide that may become necessary if weed species infect crops but weeding agribots may be a mechanical solution—based on visual recognition, they either apply herbicides in microdoses, mechanically chop weeds up or electrocute weeds at high voltage.

Animal husbandry requires considerable capital investment with highly variable and marginal return but a culturally familiar diet would comprise 20–30% meat and 70–80% vegetable. The problem of animal husbandry is the vast areas required for grass foraging required of cattle and sheep. It takes 10–20 kg of feed to produce 1 kg of beef or lamb meat. In China, smaller areas of foraging and more limited animal food choices are accommodated by adopting chickens and pigs. Pigs offer better return with 1 kg of pork meat from 5.6 kg of feed. Chickens offer a much higher return with 1 kg of chicken meat from 3.3 kg of feed. This is similar to silkworm. The use of chickens only for eggs still further reduces the farming area required. Insects which can consume vegetable waste represent a low-fat, protein-rich source of human food or as animal feed, e.g., silkworm (*Bombyx mori*), large hawkmoth (*Agrius convolvuli*) and termite (*Macrotermes subhyalinus*) [79]. Insects are biologically similar to common seafoods such as prawns. Silkworm is eaten in China as a delicacy—they are easy to cultivate and demand modest resources while producing little waste. Silkworm larvae exclusively consume (human-inedible) mulberry leaves for 25 days which requires dedicated land area—nevertheless they produce cocoon silk for other purposes. Hawkmoth pupae is much larger but the reproductive adult is airborne imposing complications of containment. Insects may be dried and ground into flour. Termites exploit and consume fungus gardens within termite mounds to indirectly consume inedible plant material—although wingless, kings and queens sprout wings when sexually mature to form new colonies presenting challenges to containment.

Aquaculture combines food production with waste treatment in an already neutral buoyancy environment. Aquatic animals (such as fish and seafood) and plants (such as seaweed) provide a compact form of animal husbandry. Fish require ~5–20 times less energy cost per protein yield than land vertebrates offering higher protein densities per unit volume [80]. Their reproductive and embryonic development appears unaffected by microgravity environments. A commonly proposed fish for aquaculture is *Tilapia* which may be cultivated in subtropical fish tanks but it has difficulties in processing complex polysaccharides. Around 500 kg of catfish can be grown an a 1 m^3^ tank per year but fish-breeding requires lighting [81]. The freshwater armoured catfish *Hoplosternum* (Hassar) is a bottom-feeding mud-dweller that inhabits low-oxygen pools and can survive drying up of pools. It can gulp air, absorbing oxygen in its gut and expelling exhaled air through its anus. It consumes benthic invertebrates, algae and detritus. They reach sexual maturity in a year and breed by forming bubble nests with serendipitous materials such as plant debris. They lay up to several hundred eggs at a time and their protective behaviour makes it easy to catch. It has a pink salmon-like flesh and is eaten curried in its armour in Guyana which is easily lifted off once cooked. It is commonly eaten with Guyanese bhaji (sauteed spinach, chili, onions, garlic and tomatoes without the fritters) providing a broad-based nutritional meal. Closed equilibrated biological aquatic system (CEBAS) comprised a four-compartment closed fully-submerged aquatic ecosystem with integrated waste management of fish (Chinese grass carp *Ctenopharyngodon idella*), water snails (*Biomphalaria glabrata*), ammonia-oxidising bacteria biofilter to convert ammonia into nitrate and edible non-gravitropic water plants (hornweed *Ceratophyllum demersum* fed to the carp) was demonstrated on the STS-89 and STS-90 flights [80]. Algae is more readily processed as waste by fish. Wastewater and air revitalisation may be implemented through algae farming with food production implemented through hydroponics.

In photosynthesis, sunlight invokes the transfer of electrons mediated by photosynthetic complexes through an organic electric circuit—one glucose molecule is synthesised per 48 photons absorbed. Photosynthesis in higher plants in converting CO_2_ into O_2_ is rather inefficient at ~1–3% but algae offer 10–15% efficiencies. Algae—average composition C_6.14_H_10.3_O_2.24_N—have higher specific photosynthetic productivity ~5–10 times than higher plants and are also more manageable. Green algae are edible with a high nutrient load and protein-rich, e.g., *Chlorella vulgaris* comprises 40–60% protein (all amino acids), 20% carbohydrate, 10–20% fat, 15% water and almost all essential vitamins, minerals and fatty acids. However, algae are unpalatable and it is recommended that it comprise no more than 20–25% of the human diet to prevent excess protein. Seaweed is a fast-growing vegetable grown in ocean waters relieving pressure on arable land and offering high nutrient density food—3D printing of seaweed allows combination with more palatable flavours for bulk consumption. Seaweed is a high protein alga that is commonly consumed as laverbread in Wales. Populations of photosynthetic cyanobacteria or purple non-sulphur bacteria may be cultivated to produce biosynthesised organic nutrients and medicines from solar energy and waste CO_2_ [82]—oxygen, proteins, vitamins, sugars, antibiotics, etc. A single human releases 1 kg CO_2_/day. At 1 AU, solar flux is 8.6 × 10^25^ photons/m^2^/day with a microbial biomass production rate of 2.64 × 10^25^ photons/kg glucose, i.e., 1.5 kg glucose/m^2^/day. Cyanobacteria are particularly suited to Mars [83] but also suited to the Moon. *Arthrospira* (*Spirulina*) is a filamentous cyanobacteria that is easily cultured at 28–32 °C and sun-dried into cakes for its high nutritional value (though insufficient vitamin C) [84]) but also offers high photosynthetic efficiency in generating oxygen some 2.5–4.0 times more productively than trees. Spirulina offers many advantages over Chlorella including easier harvesting, ready digestibility and resistance to microbial infection [85]. Spirulina is a rich source of protein (~70% by mass) and photosynthetic oxygen (0.5 kg algae consumes ~2 kg CO_2_) [86]. A photo-bioreactor that grew Spirulina has demonstrated the feasibility of algae as part of CELSS [87]. The addition of taste proteins makes nutritional bacteria more palatable, e.g., brazzein (sweetness), miraculin (sweet from sour), valencene (oranges), etc. Synthetic biology may be employed to enhance the productivity of Spirulina by utilising non-CO_2_ inputs but any organic can be readily oxidised into CO_2_ rendering this option redundant [88].

## 7. Robotics and Automation

A typical crew 24-h day might comprise 8.5 h sleep, 1.5 h personal and habitat cleaning, 3 h communal meal breaks, 8 h work, 2 h exercise and 1 h personal time. Given the tight work schedule, it is of high importance to automate as much habitat activities as possible. It is expected that robotics will play a significant role within the lunar base to relieve astronaut workloads. Robotics and automation in agriculture will have the largest impact on astronaut workload as this involves monitoring large-area crop status and the ability to react to enhance crop productivity to yield high quality, healthy crops. This will require large-scale persistent environmental monitoring using sensor networks in a challenging environment [89]. Data muling involves collecting data from fixed sensor nodes as the mobile rover passes within communication range of the node. Environmental variables to be monitored—temperature, pressure, airflow, light intensity, pH and biochemical sensing. Rather than complex molecular analytic instrumentation, biochemical sensing can be implemented using electrochemical cells, turbidity nephelometry, cytometers, and gas microsensors though with diminished capability. The employment of distributed sensing permits the employment of multisensor Kalman filters to robustify estimates. Agricultural measurements [90] include monitoring of soil parameters—moisture (electrical conductivity), pH, compaction (strain gauge-based mechanical impedance) and nitrogen/carbon load (near infrared spectroscopy)—and monitoring of vegetation status—weed control (visual identification), crop maturity through sugar and acid content (near-infrared imaging) and crop health through normalised difference vegetation index (multispectral imaging). The robotics aspect requires sophisticated autonomous tractors in coordinated multirobot teams capable of following complex paths through a crop field map constructed via visual navigation as well as complex interaction with the crops such as seeding, cutting, grafting, transplanting, weeding, harvesting, etc. Hydroponics eases the complexity of some of these requirements but seeding, transporting, transplanting and harvesting are still complex robotic processes. Bioreactors that are employed extensively in CELSS require autonomous monitoring and control of multiple physical and biochemical parameters—medium temperature, photosynthetic active radiation exposure, pumped fluid flow circulation rate, motorised fluid stirring rate, gas input valve flow rate (NO_x_ and CO_2_), nephelometric cell density (microbial growth), medium pH levels [91]. Inherent time delays and complex dependencies in the bioreactor impose the requirement for intelligent feedback control with feedforward predictive capability.

## 8. Discussion

The food and revitalisation module (FARM) greenhouse of 528 m^2^ implementing a range of 21 crops supplies 100% of human nutritional requirements for 18 astronauts [92]. A simulated bioregenerative life support system supplemented by physicochemical methods (for atmospheric recycling, water recycling, waste reclamation and food production) with 15 types of hydroponic crop, silkworm husbandry and both solid and liquid waste recovery yielded 29.7 kg of oxygen, potable and hygiene water and food at 2700 calories/person/day (375.5 g carbohydrate, 99.5 g protein and 91.2 g fat) with 99.4% material closure [93]. However, caloric restriction of energy intake by 20–40% to 1600 calories/day (met with a diet of 3/2 cups of wheat, ½ cup of soya beans, ½ cup of pinto beans, 1 stalk of broccoli, ½ cup of spinach, ¼ cup of peanuts and a small amount of mushrooms) can potentially reduce the incidence of cancer to offset radiation exposure [94] as long as micronutrients (carotenoids, etc) diet is maintained as antioxidants to DNA damage [95] (evolved as an evolutionary strategy of postponed reproductive investment in favour of temporary somatic maintenance [96]). Although such a draconian diet may foster physiological advantages, it is unlikely to foster psychological well-being.

Flight experiments of cropping in space have generated mixed results including diminutive or abnormal crops, lack of seed production, etc which have yet to be resolved but it is unclear if these problems would persist under partial gravity. Based on experimental and theoretical data from MELISSA and BIOS, near closed loop mass flows were established for a bioregenerative life support system to support a crew of six for a 780-day roundtrip Mars mission [97]. They concluded that the CELSS mass would be 18.09 tonnes (3 tonnes/crew member), some four times higher than an expanded ISS-type physicochemical life support system of 4.83 tonnes (while the latter incorporated double redundancy, the former did not). The system however was oxygen-deficient but this could be supplied from in-situ electrolysis of local water ice. Hence, bioregenerative systems require very high initial mass for plant growth and other supporting equipment and are suitable only for very long duration missions to avoid the cost of launching large amounts of food supplies over multiple years (revised to mass at 53.75 tonne-years per person) [98]. The high system mass and volume of extra-terrestrial food production beyond physicochemical methods becomes cost-effective only for missions lasting in excess of 2–3 years. Much of this is attributable to their high-power consumption and large buffering volumes to compensate for long response lags. The amount of crop required to feed one person is around 200% that required to supply oxygen and water for one person—hence, growing 50% of the food requirement is considered to be the most efficient approach. Hence, 100% food production would produce 100% excess water beyond requirements which could be diverted to propellant/oxidant production. Staple carbohydrate crops such as wheat, potato and rice are more efficient than growing protein and fat crops such as soyabeans and peanuts. Rice unfortunately requires considerable agricultural area—around 20.1 m^2^/person compared with 6.6 m^2^/person for wheat (Table 2 in [93]). However, this analysis is only applicable if all the bioregenerative equipment is launched from Earth—much of it can be built in-situ from in-situ resources [11]. There are sustainability lessons in developing CELSS that can be applied to Earth’s biosphere through recycling and regeneration [99,100]. The author concurs but suggests that it is a two-way process and that extra-terrestrial settlement must be sustainable:

1. Maximise exploitation of renewable energy sources (i.e., solar energy) and minimise consumption of non-renewable energy sources (i.e., H_2_/O_2_ combustion)
2. Minimise the generation of toxic by-products, e.g., Cr;
3. Develop industrial ecosystems of interlocking processes that feed waste of one process into another;
4. Exploit feedback loops to recycle scarce resources.

It has even been suggested that CELSS technology such as MELiSSA may assist in reclaiming hot desert regions to counter rapid desertification for productive arable farming using closed resource cycles [101]. Such regions offer high intensity PAR (productivity increases linearly with photo intensity until saturation at 2.5L_0_), high temperature (by 30% from 17 °C to 23 °C) and high atmospheric CO_2_ levels (by 30–40% at 800 ppm) to enhance arable productivity. Experiments in the Laboratory Biosphere and elsewhere suggest that elevated CO_2_ concentrations up to 2000 ppm enhance crop productivity proportionally [102,103]. This can be implemented in a lunar CELSS system provided high carbon recycling can be implemented. Nitrogen as a buffer gas is the chief limitation as it is scarce on the Moon and rarefied at <5% of atmospheric composition on Mars [104]. Crucial to the realisation of robust human habitation of extra-terrestrial environments such as the Moon will be in recycling of scarce nutrients CNPSK etc through CELSS technology supplemented with an industrial ecology that can supply a restricted set of indigenous elements.

## Figures and Tables

**Table 1:** LCROSS ejecta plume show the paucity of volatile species [8].

Volatile Species.	% Relative to Water	% by Mass
H_2_O	100	5.60
H_2_S	16.75	0.94
NH_3_	6.03	0.34
SO_2_	3.19	0.18
C_2_H_4_	3.12	0.17
CO_2_	2.17	0.12
CH_3_OH	1.55	0.09
CH_4_	0.65	0.04
OH	0.03	0.002

## Data Availability

Not applicable.
